# Supplementary material for: Bridging the Gap Between Platforms: Comparing Grape Phylloxera Daktulosphaira vitifoliae (Fitch) Microsatellite Allele Size and DNA Sequence Variation
Source: Insects. 2025 Feb 19;16(2):230. doi: 10.3390/insects16020230 (PMC11856487; doi:10.3390/insects16020230)
Supplement: Supplementary file 1 [file insects-16-00230-s001.zip › insects-3394970-supplementary.pdf]

Supplementary Figure S1. Reported size range of alleles (allelic diversity) and size overlap between phylloxera SSR loci reported in Corrie *et al.* (2002), Lin *et al.* (2006), Vorwerk & Forneck (2006), and Umina *et al.* (2007). Grey shading indicates loci selected as preferred standard markers in the current study, the fluorescent label and universal tail (*sensu* Blacket *et al.* 2012) employed in multiplex PCR reactions in the present study is indicated above alleles. Minimum reported allele sizes are shown as squares, maximum sizes as triangles.

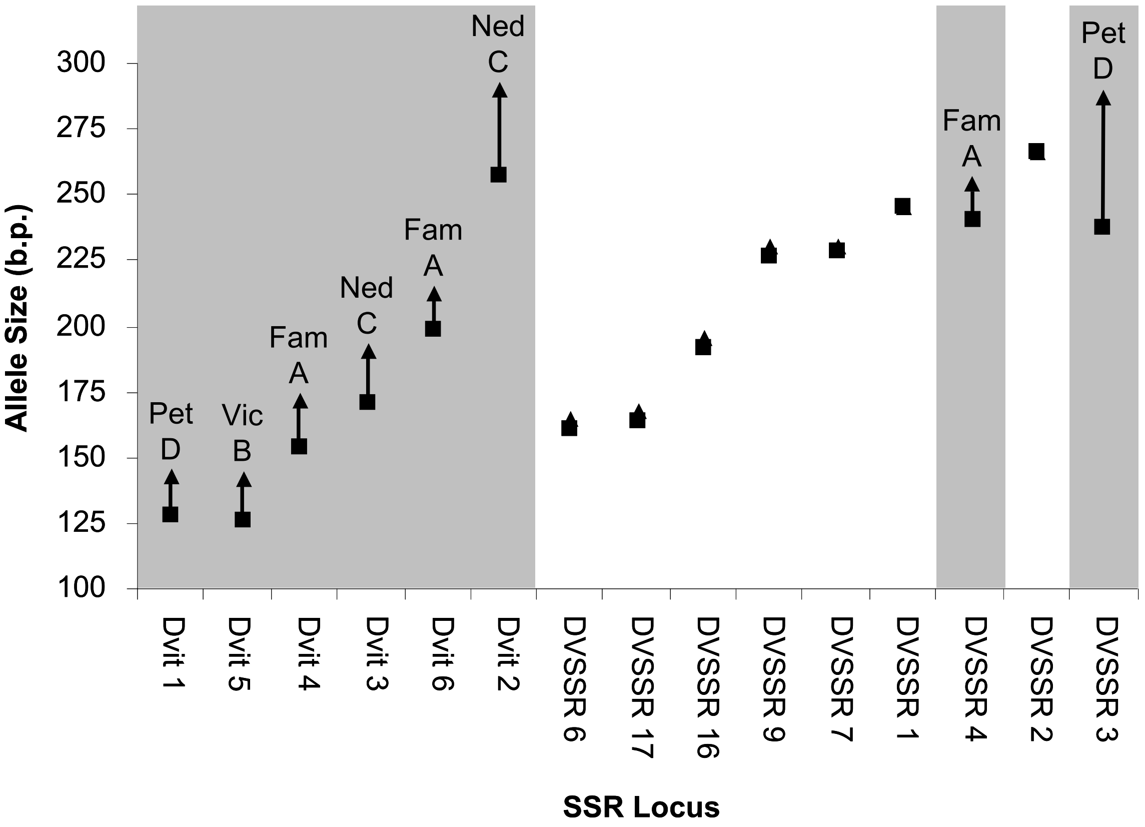

Supplementary Figure S2. Size and frequency of SSR alleles represented in Australian genotypes (Dvit loci, Umina *et al.* 2007), or European/Californian (DVSSR loci, Lin *et al.* 2006) genotypes of phylloxera. Numbers indicate allele sizes in base pairs. Each dot represents an allele present in a known genotype.

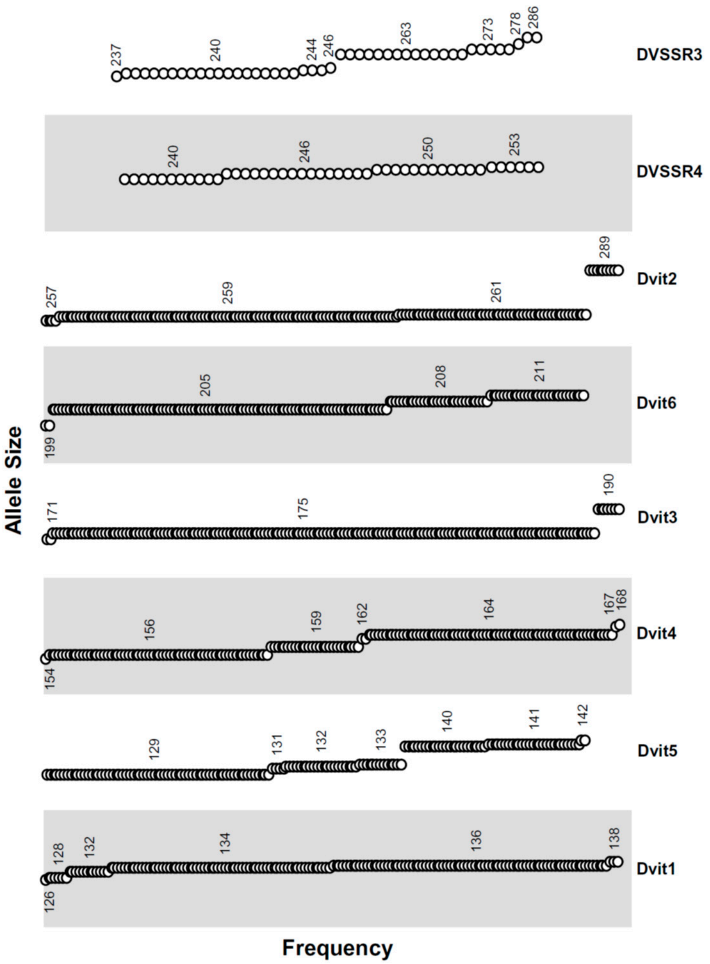

Supplementary Figure S3. Alignments of allele HTS sequences from DVIT1. Forward and reverse primer binding positions annotated in green and blue. DNA bases represented by G = yellow, A = red, T = green, C = blue.

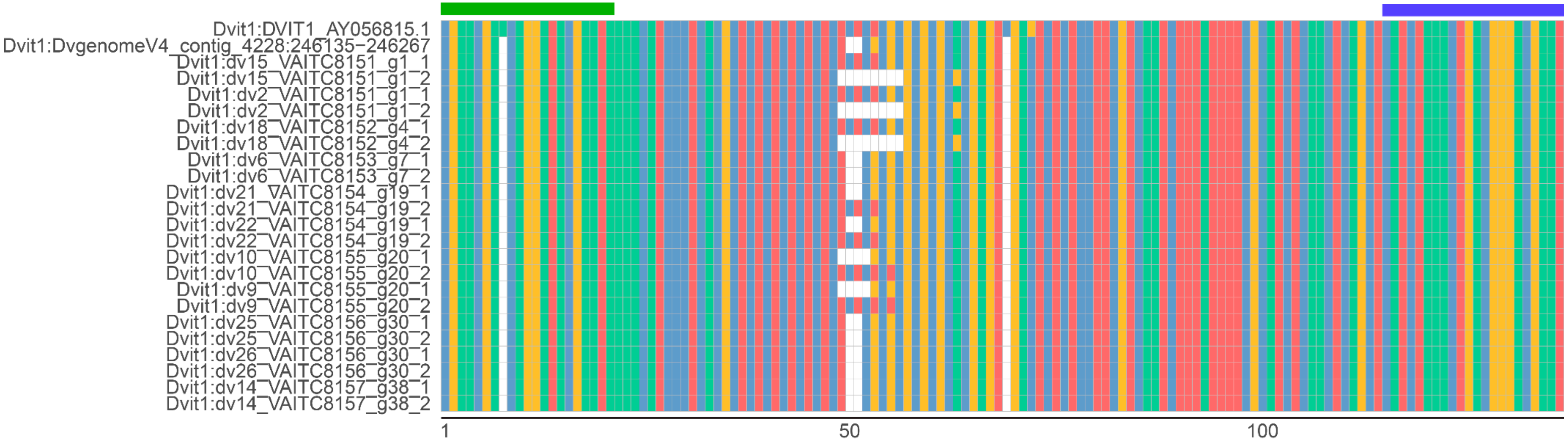

DVIT1 Alleles

PQ788553 DVIT1:dv15_VAITC8151_g1_1_134bp

CGTTCGTCTGGTATCGTTATTTTCTACCCACTCGCACACACACACACACACACGCGCGCTCGTGAGTCACACACCAACGACTTACAAAT
AAAACGCTACACTTCACTGCACTACATTTCAGTCGGGTCGTTA

PQ788554 DVIT1:dv15_VAITC8151_g1_2_126bp

CGTTCGTCTGGTATCGTTATTTTCTACCCACTCGCACACACACACACGCGCGCGCGTGAGTCACACACCAACGACTTACAAATAAAACGCT
ACACTTCACTGCACTACATTTCAGTCGGGTCGTTA

PQ788555 DVIT1:dv6_VAITC8153_g7_1_132bp

CGTTCGTCTGGTATCGTTATTTTCTACCCACTCGCACACACACACACACGCGCGCGCTCGTGAGTCACACACCAACGACTTACAAATAA
AACGCTACACTTCACTGCACTACATTTCAGTCGGGTCGTTA

PQ788556 DVIT1:dv10_VAITC8155_g20_1_130bp

CGTTCGTCTGGTATCGTTATTTTCTACCCACTCGCACACACACACACGCGCGCGCTCGTGAGTCACACACCAACGACTTACAAATAAAA
CGCTACACTTCACTGCACTACATTTCAGTCGGGTCGTTA

PQ788557 DVIT1:dv10_VAITC8155_g20_2_134bp

CGTTCGTCTGGTATCGTTATTTTCTACCCACTCGCACACACACACACACACACGCGCGCTCGTGAGTCACACACCAACGACTTACAAAT
AAAACGCTACACTTCACTGCACTACATTTCAGTCGGGTCGTTA

Supplementary Figure S4. Alignments of allele HTS sequences from DVIT2. Forward and reverse primer binding positions annotated in green and blue. DNA bases represented by G = yellow, A = red, T = green, C = blue.

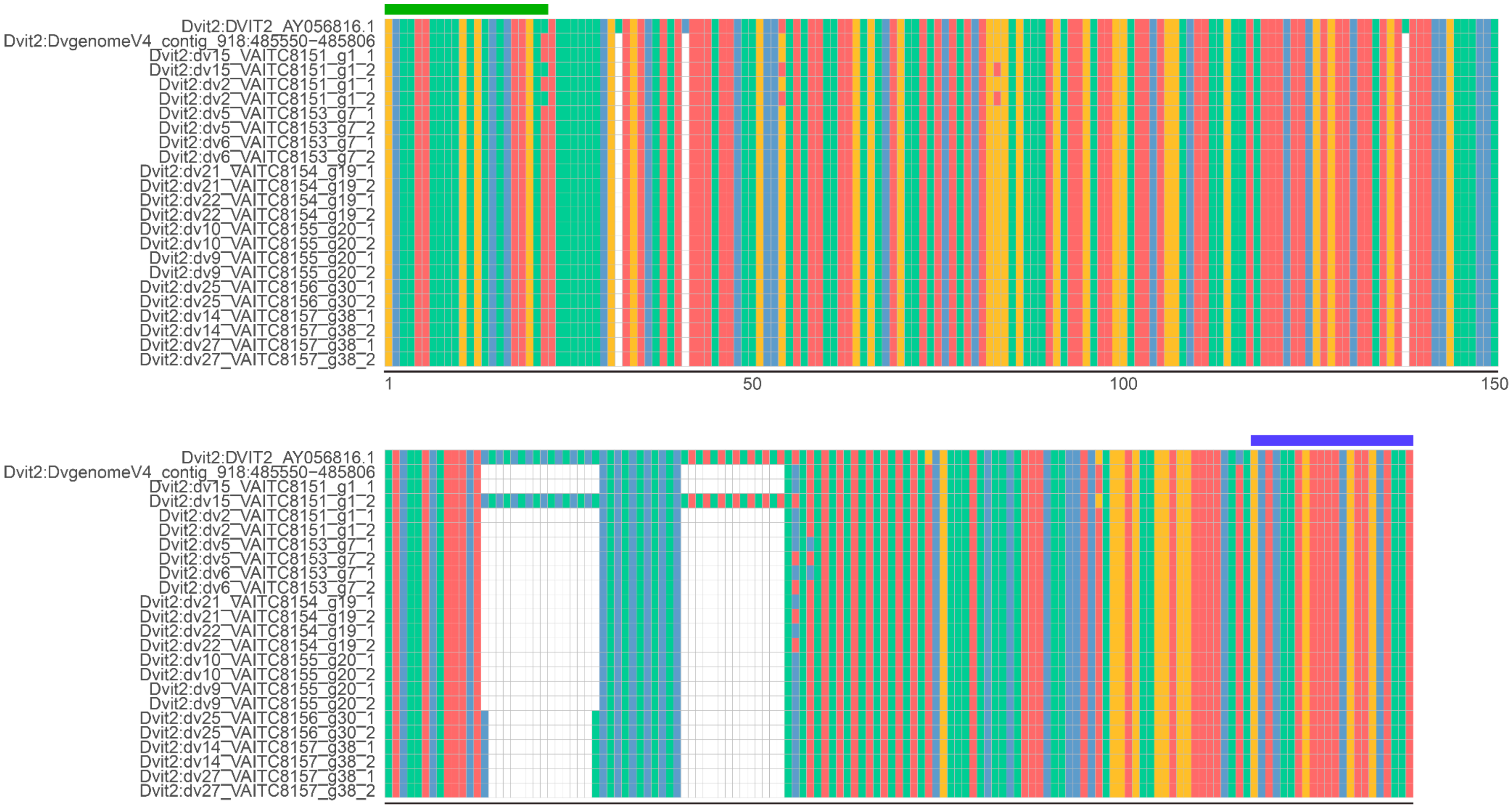

DVIT2 Alleles

PQ788558 DVIT2:dv15_VAITC8151_g1_1_256bp

GCTTAATTTTGTGTCTCAAGTAATTTTTTCGAGACTATAAAATAACTTGCCGTATAATTAAGTGTCAGTTATACATACAGGGTGTTTAGTAAGT
AAGGTAACAGGTCAATTGTTATAAACAACGAGAACAATAGAAAACCGTTTCCTTACTTACTAAACACTCTCTCTCTCTCTATATATATATATATAT
ATACGTTTATCTTCTAAACTTCCACATGGAGTTGGAGGAAAACTATGCACTTAGAAAACGAAGCATTA

PQ788559 DVIT2:dv15_VAITC8151_g1_2_286bp

GCTTAATTTTGTGTCTCAAGTTATTTTTTCGAGACTATAAAATAACTTGCCATATAATTAAGTGTCAGTTATACATACAGAGTGTTTAGTAAGT
AAGGTAACAGGTCAATTGTTATAAACAACGAGAACAATAGAAAACCGTTTCCTTACTTACTAAACACTCTCTCTCTCTCTCTCTCTCTCTCTCT
ATATATATATATATATATATATATATATATATATACGTTTATCTTCTAAACTTCCACGTGGAGTTGGAGGAAAACTATGCACTTAGAAAACGAAGC
ATTA

PQ788560 DVIT2:dv2_VAITC8151_g1_2_256bp

GCTTAATTTTGTGTCTCAAGTTATTTTTTCGAGACTATAAAATAACTTGCCATATAATTAAGTGTCAGTTATACATACAGAGTGTTTAGTAAGT
AAGGTAACAGGTCAATTGTTATAAACAACGAGAACAATAGAAAACCGTTTCCTTACTTACTAAACACTCTCTCTCTCTCTATATATATATATATAT
ATACGTTTATCTTCTAAACTTCCACATGGAGTTGGAGGAAAACTATGCACTTAGAAAACGAAGCATTA

PQ788561 DVIT2:dv5_VAITC8153_g7_1_256bp

GCTTAATTTTGTGTCTCAAGTAATTTTTTCGAGACTATAAAATAACTTGCCGTATAATTAAGTGTCAGTTATACATACAGGGTGTTTAGTAAGT
AAGGTAACAGGTCAATTGTTATAAACAACGAGAACAATAGAAAACCGTTTCCTTACTTACTAAACACTCTCTCTCTCTCTATATATATATATATAT
ATACGTTTATCTTCTAAACTTCCACATGGAGTTGGAGGAAAACTATGCACTTAGAAAACGAAGCATTA

PQ788562 DVIT2:dv5_VAITC8153_g7_2_256bp

GCTTAATTTTGTGTCTCAAGTAATTTTTTCGAGACTATAAAATAACTTGCCGTATAATTAAGTGTCAGTTATACATACAGGGTGTTTAGTAAGT
AAGGTAACAGGTCAATTGTTATAAACAACGAGAACAATAGAAAACCGTTTCCTTACTTACTAAACACTCTCTCTCTCTATATATATATATATATAT
ATACGTTTATCTTCTAAACTTCCACATGGAGTTGGAGGAAAACTATGCACTTAGAAAACGAAGCATTA

PQ788563 DVIT2:dv25_VAITC8156_g30_1_258bp

GCTTAATTTTGTGTCTCAAGTAATTTTTTCGAGACTATAAAATAACTTGCCGTATAATTAAGTGTCAGTTATACATACAGGGTGTTTAGTAAGT
AAGGTAACAGGTCAATTGTTATAAACAACGAGAACAATAGAAAACCGTTTCCTTACTTACTAAACACTCTCTCTCTCTCTATATATATATATAT
ATATACGTTTATCTTCTAAACTTCCACATGGAGTTGGAGGAAAACTATGCACTTAGAAAACGAAGCATTA

Supplementary Figure S5. Alignments of allele HTS sequences from DVIT3. Forward and reverse primer binding positions annotated in green and blue. DNA bases represented by G = yellow, A = red, T = green, C = blue.

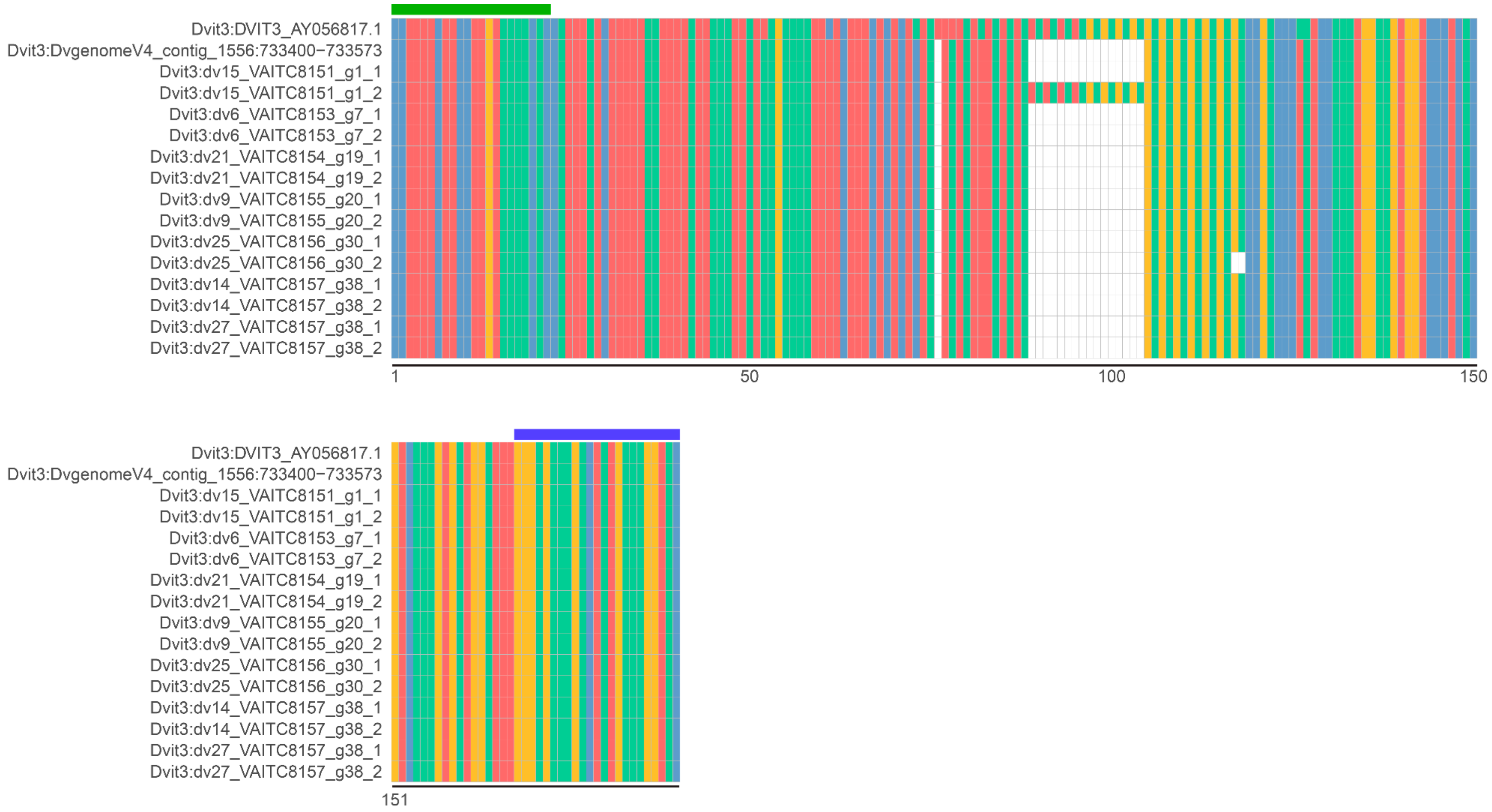

DVIT3 Alleles

PQ788564 DVIT3:dv15_VAITC8151_g1_1_173bp

CCAAAACAACCAAGATTTTCTCCTAAATACAAAAATTAAAATAATTTAATATTGTTTTAAAACAAACACACACATATATAAATATATGTGTGTGT
GTGTGTCCGTCCCATACCTTTAGGTTGAGGACCCACTCGACTTTGAGTAGGTAAAGGGTGTTTGTCATAGTTTGGATC

PQ788565 DVIT3:dv15_VAITC8151_g1_2_189bp

CCAAAACAACCAAGATTTTCTCCTAAATACAAAAATTAAAATAATTTAATATTGTTTTAAAACAAACACACACATATATAAATATATATATATAT
GTGTGTGTGTGTGTGTGTGTGTCCGTCCCATACCTTTAGGTTGAGGACCCACTCGACTTTGAGTAGGTAAAGGGTGTTTGTCATAGTTTGG
ATC

PQ788566 DVIT3:dv25_VAITC8156_g30_2_171bp

CCAAAACAACCAAGATTTTCTCCTAAATACAAAAATTAAAATAATTTAATATTGTTTTAAAACAAACACACACATATATAAATATATGTGTGTGT
GTGTCCGTCCCATACCTTTAGGTTGAGGACCCACTCGACTTTGAGTAGGTAAAGGGTGTTTGTCATAGTTTGGATC

Supplementary Figure S6. Alignments of allele HTS sequences from DVIT4. Forward and reverse primer binding positions annotated in green and blue. DNA bases represented by G = yellow, A = red, T = green, C = blue.

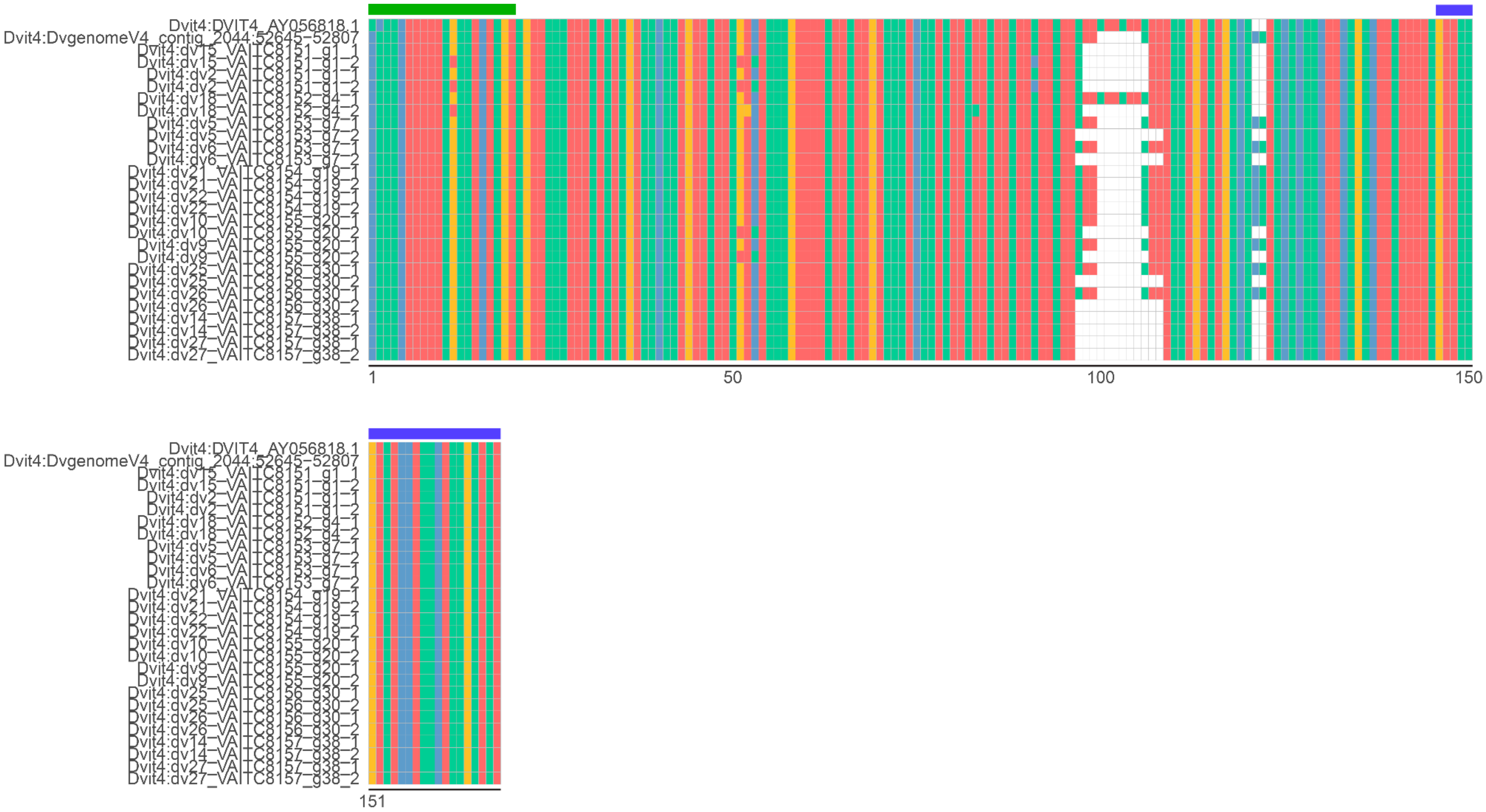

DVIT4 Alleles

PQ788567 DVIT4:dv15_VAITC8151_g1_1_157bp

CTTTCAAAAATGTTACATGATGAATTTAAATATATGATTCTTAGAATAATGACATTTGAAAATAATAAGATTTACTTATAATAATAATAATAATAA TAAATTAGATAGTCTATCTCTTCAACTGTCAATAAAATGAATTGATACCATTCATTGTATA

PQ788568 DVIT4:dv15_VAITC8151_g1_2_157bp

CTTTCAAAAATATTACATGATGAATTTAAATATATGATTCTTAGAATAATAATATTTGAAAATAATAAGATTTACTTATAATAATAATAACAATAA TAAATTAGATAGTCTATCTCTTCAACTGTCAATAAAATGAATTGATACCATTCATTGTATA

PQ788569 DVIT4:dv18_VAITC8152_g4_1_166bp

CTTTCAAAAATGTTACATGATGAATTTAAATATATGATTCTTAGAATAATGACATTTGAAAATAATAAGATTTACTTATAATAATAATAATAATAA TAATAATAATAAATTAGATAGTCTATCTCTTCAACTGTCAATAAAATGAATTGATACCATTCATTGTATA

PQ788570 DVIT4:dv18_VAITC8152_g4_2_157bp

CTTTCAAAAATATTACATGATGAATTTAAATATATGATTCTTAGAATAATGGCATTTGAAAATAATAAGATTTACTTATAATTATAATAATAATAA TAAATTAGATAGTCTATCTCTTCAACTGTCAATAAAATGAATTGATACCATTCATTGTATA

PQ788571 DVIT4:dv5_VAITC8153_g7_1_162bp

CTTTCAAAAATGTTACATGATGAATTTAAATATATGATTCTTAGAATAATGACATTTGAAAATAATAAGATTTACTTATAATAATAATAATAATAA TAATAAATTAGATAGTCTCTATCTCTTCAACTGTCAATAAAATGAATTGATACCATTCATTGTATA

PQ788572 DVIT4:dv5_VAITC8153_g7_2_154bp

CTTTCAAAAATGTTACATGATGAATTTAAATATATGATTCTTAGAATAATGACATTTGAAAATAATAAGATTTACTTATAATAATAATAATAATAA ATTAGATAGTCTATCTCTTCAACTGTCAATAAAATGAATTGATACCATTCATTGTATA

PQ788573 DVIT4:dv10_VAITC8155_g20_2_157bp

CTTTCAAAAATGTTACATGATGAATTTAAATATATGATTCTTAGAATAATAATATTTGAAAATAATAAGATTTACTTATAATAATAATAATAATAA TAAATTAGATAGTCTATCTCTTCAACTGTCAATAAAATGAATTGATACCATTCATTGTATA

Supplementary Figure S7. Alignments of allele HTS sequences from DVIT6. Forward and reverse primer binding positions annotated in green and blue. DNA bases represented by G = yellow, A = red, T = green, C = blue.

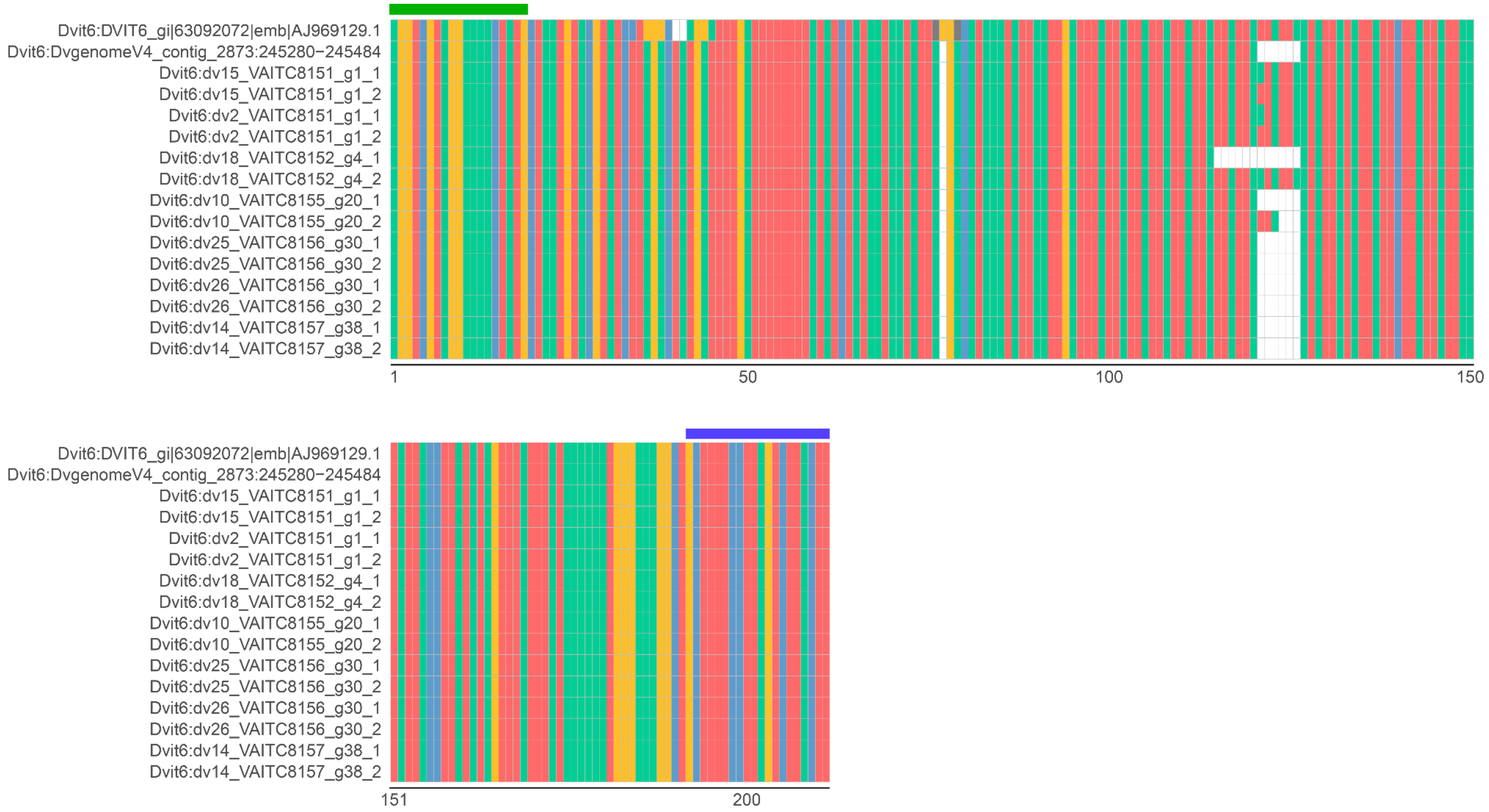

DVIT6 Alleles

PQ788574 DVIT6:dv15_VAITC8151_g1_1_210bp

TGGACGATGGTTTTCATAGCATTAGATCGATACAATGTCATAGTAAAAGTAAAAAAAATATACATATTATTATAATGTCTATTTATAATTAAGTA
AATAATAATAATAATAATAATAATTATAATTATAATATAATAACAATAATAATTATAATCCAATATATGAAATAAATATTTTTTAGGGTTTGGCAG
CAAAACCAATGACAATCAA

PQ788575 DVIT6:dv15_VAITC8151_g1_2_210bp

TGGACGATGGTTTTCATAGCATTAGATCGATACAATGTCATAGTAAAAGTAAAAAAAATATACATATTATTATAATGTCTATTTATAATTAAGTA
AATAATAATAATAATAATAATAATAATAATTATAATATAATAACAATAATAATTATAATCCAATATATGAAATAAATATTTTTTAGGGTTTGGCAG
CAAAACCAATGACAATCAA

PQ788576 DVIT6:dv18_VAITC8152_g4_1_198bp

TGGACGATGGTTTTCATAGCATTAGATCGATACAATGTCATAGTAAAAGTAAAAAAAATATACATATTATTATAATGTCTATTTATAATTAAGTA
AATAATAATAATAATAATTATAATATAATAACAATAATAATTATAATCCAATATATGAAATAAATATTTTTTAGGGTTTGGCAGCAAAACCAATG
ACAATCAA

PQ788577 DVIT6:dv10_VAITC8155_g20_1_204bp

TGGACGATGGTTTTCATAGCATTAGATCGATACAATGTCATAGTAAAAGTAAAAAAAATATACATATTATTATAATGTCTATTTATAATTAAGTA
AATAATAATAATAATAATAATAATTATAATATAATAACAATAATAATTATAATCCAATATATGAAATAAATATTTTTTAGGGTTTGGCAGCAAAAC
CAATGACAATCAA

PQ788578 DVIT6:dv10_VAITC8155_g20_2_207bp

TGGACGATGGTTTTCATAGCATTAGATCGATACAATGTCATAGTAAAAGTAAAAAAAATATACATATTATTATAATGTCTATTTATAATTAAGTA
AATAATAATAATAATAATAATAATAATTATAATATAATAACAATAATAATTATAATCCAATATATGAAATAAATATTTTTTAGGGTTTGGCAGCAA
AACCAATGACAATCAA

Supplementary Figure S8. Alignments of allele HTS sequences from DVSSR3. Forward and reverse primer binding positions annotated in green and blue. DNA bases represented by G = yellow, A = red, T = green, C = blue.

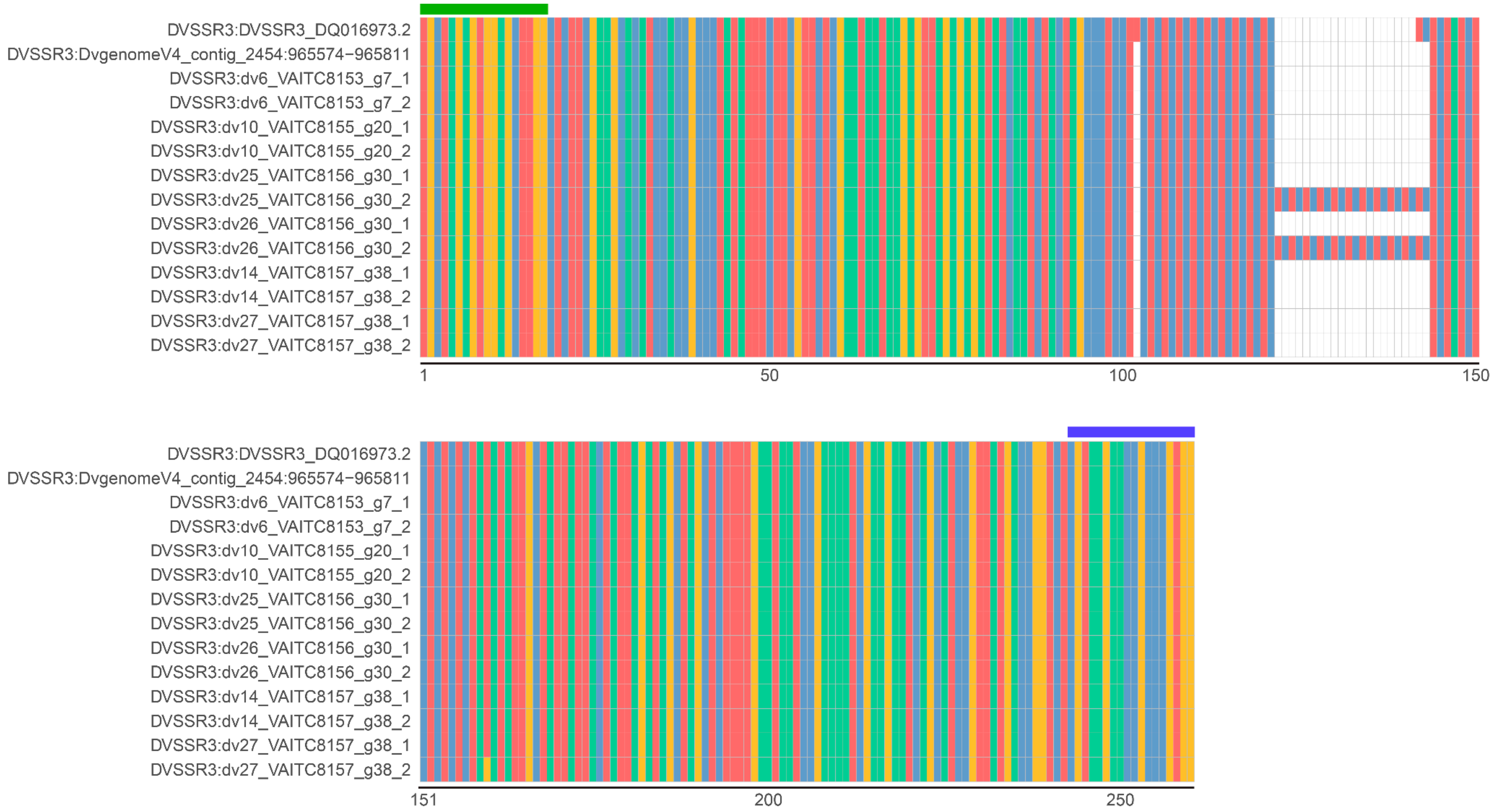

DVSSR3 Alleles

PQ788579 DVSSR3:dv6_VAITC8153_g7_1_237bp

AGCATGTGAGGTGCAAGGCACAACGTTGCTCTACCTCCGCCCATATAAACAACGAACACGTTATTATTGTGAATGTGTGTATACTTACATCA
TGCCCACCACACACACACACACACACACACATACACACACACATATATAAGCATAATAATCATAATGTATGCATGCACAAAAGTTATTACCGT
TTTACGTTGTTACTGCTACCGAATAGTCCGGACACGATTGTTCCGCCCGAGG

PQ788580 DVSSR3:dv25_VAITC8156_g30_2_259bp

AGCATGTGAGGTGCAAGGCACAACGTTGCTCTACCTCCGCCCATATAAACAACGAACACGTTATTATTGTGAATGTGTGTATACTTACATCA
TGCCCACCACACACACACACACACACACACACACACACACACACACACACACATACACACACACATATATAAGCATAATAATCATAATGTAT
GCATGCACAAAAGTTATTACCGTTTTACGTTGTTACTGCTACCGAATAGTCCGGACACGATTGTTCCGCCCGAGG

PQ788581 DVSSR3:dv27_VAITC8157_g38_2_237bp

AGCATGTGAGGTGCAAGGCACAACGTTGCTCTACCTCCGCCCATATAAACAACGAACACGTTATTATTGTGAATGTGTGTATACTTACATCA
TGCCCACCACACACACACACACACACACACATACACACACACATGTATAAGCATAATAATCATAATGTATGCATGCACAAAAGTTATTACCG
TTTTACGTTGTTACTGCTACCGAATAGTCCGGACACGATTGTTCCGCCCGAGG

OFFICIAL

Supplementary Figure S9. Alignments of allele HTS sequences from DVSSR4. Forward and reverse primer binding positions annotated in green and blue. DNA bases represented by G = yellow, A = red, T = green, C = blue.

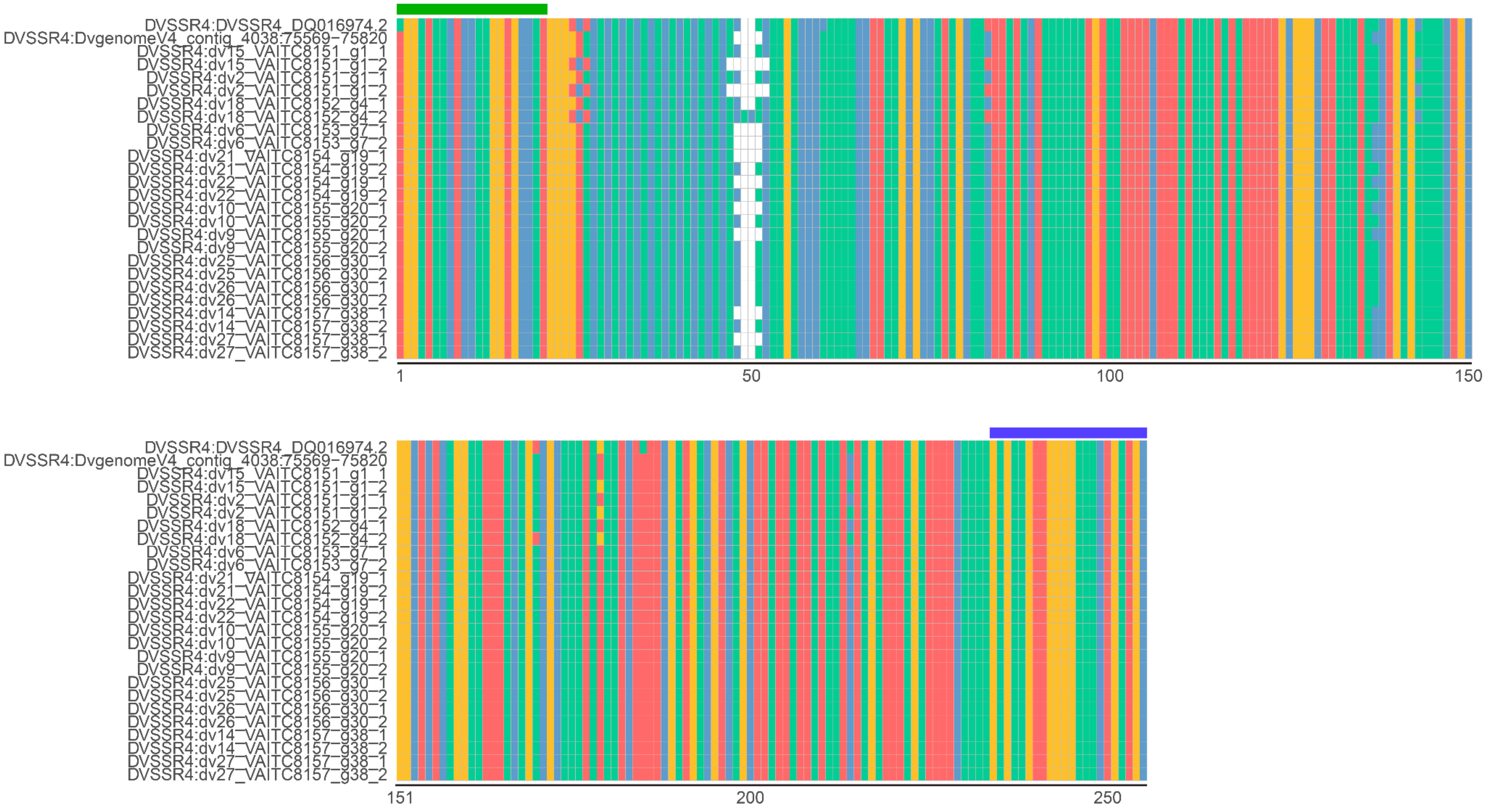

OFFICIAL

DVSSR4 Alleles

PQ788582 DVSSR4:dv15_VAITC8151_g1_1_253bp

AGGTATTCACCTTGGAGCCTAGGGGATCTCTCTCTCTCTCTCTCTCTCTTGTCCCTTTTTCCAATTGCGCCTATGCTTCAATATCATTTTTT
AGATTAAAACAAATATTTATATAAAAAGCGGGCAATTTATTCAGTGTTTTCAGCGGCACACTGGTTAAATCTGTCGCTTTATATTACAAAACGT
AGTCGACTGCAATAATAATATTACATGTAAATGTAAAACTTTTGTGTTGAAGGGGTTTCAGTAGC

PQ788583 DVSSR4:dv15_VAITC8151_g1_2_249bp

AGGTATTCACCTTGGAGCCTAGGGACACTCTCTCTCTCTCTCTCTCTTGTCCCTTTTTCCAATTGCGCCTATGCTTAAATATCATTTTTTAGAT
TAAAACAAATATTTATATAAAAAGCGGGCAATTTATTCAGTGCTTTCAGCGGCACACTGGTTAAATCTGTCGCTTTATGTTACAAAACGTAGT
CGACTGCAATAATAATATTATATGTAAATGTAAAACTTTTGTGTTGAAGGGGTTTCAGTAGC

PQ788584 DVSSR4:dv18_VAITC8152_g4_2_255bp

AGGTATTCACCTTGGAGCCTAGGGACACTCTCTCTCTCTCTCTCTCTCTCTCTTGTCCCTTTTTCCAATTGCGCCTATGCTTAAATATCATTT
TTTAGATTAAAACAAATATTTATATAAAAAGCGGGCAATTTATTCAGTGCTTTCAGCGGCACACTGGTTAAATCTGACGCTTTATGTTACAAAA
CGTAGTCGACTGCAATAATAATATTATATGTAAATGTAAAACTTTTGTGTTGAAGGGGTTTCAGTAGC

PQ788585 DVSSR4:dv6_VAITC8153_g7_1_251bp

AGGTATTCACCTTGGAGCCTAGGGGATCTCTCTCTCTCTCTCTCTCTCTTGTCCCTTTTTCCAATTGCGCCTATGCTTCAATATCATTTTTTA
GATTAAAACAAATATTTATATAAAAAGCGGGCAATTTATCCAGTGTTTTCAGCGGCACACTGGTTAAATCTGTCGCTTTATATTACAAAACGT
AGTCGACTGCAATAATAATATTACATGTAAATGTAAAACTTTTGTGTTGAAGGGGTTTCAGTAGC

PQ788586 DVSSR4:dv14_VAITC8157_g38_2_253bp

AGGTATTCACCTTGGAGCCTAGGGGATCTCTCTCTCTCTCTCTCTCTCTCTTGTCCCTTTTTCCAATTGCGCCTATGCTTCAATATCATTTTTT
AGATTAAAACAAATATTTATATAAAAAGCGGGCAATTTATCCAGTGTTTTCAGCGGCACACTGGTTAAATCTGTCGCTTTATATTACAAAACG
TAGTCGACTGCAATAATAATATTACATGTAAATGTAAAACTTTTGTGTTGAAGGGGTTTCAGTAGC

Supplementary Text S1*. *PCR primers and amplification conditions employed in multiplex PCR reactions.

PCR Primers:

Forward primer tails are underlined (below). Universal tail names refer to Blacket et al. 2012.

Primers for (FAM) Multiplex 1: (Dvit4:Dvit6:DVSSR4, 1:2:1 relative primer ratios)

Dvit4_F_Tail_A 5'-GCCTCCCTCGCGCCATCTTCAAAAATGTTACATGAT-3' (Corrie et al. 2002)

Dvit6_F_Tail_A 5'-GCCTCCCTCGCGCCATGGACGATGGTTTTCATAGC-3' (Vorwerk & Forneck 2006)

DVSSR4_F_Tail_A 5'-GCCTCCCTCGCGCCATGGTATTCACCTTGGAGCCTAG-3' (Lin et al. 2006)

Dvit4_R 5'-TATACAATGAATGGTATCAATTC-3' (Corrie et al. 2002)

Dvit6_R 5'-TTGATTGTCATTGGTTTTGC-3' (Vorwerk & Forneck 2006)

DVSSR4_R 5'-GCTACTGAAACCCCTTCAACAC-3' (Lin et al. 2006)

Tail A (FAM) (universal fluorescently labelled primer) 5'-FAM-GCCTCCCTCGCGCCA-3' (Blacket et al. 2012)

Primers for (PET) Multiplex 2: (Dvit1:DVSSR3, 1:2 relative primer ratios)

Dvit1_F_Tail_D 5'-CGGAGAGCCGAGAGGTGCGTTCGTTCTGGTATGGTTATT-3' (Corrie et al. 2002)

DVSSR3_F_Tail_D 5'-CGGAGAGCCGAGAGGTGAGCATGTGAGGTGCAAGGC-3' (Lin et al. 2006)

Dvit1_R 5'-TAACGACCCGACTGAAATGTAG-3' (Corrie et al. 2002)

DVSSR3_R 5'-CCTCGGGCGGAACAATCG-3' (Lin et al. 2006)

Tail D (PET) (universal fluorescently labelled primer) 5'-PET-CGGAGAGCCGAGAGGTG-3' (Blacket et al. 2012)

Primers for (VIC) Multiplex 3: (Single locus)

Dvit5_F_Tail_B 5'-GCCTTGCCAGCCCGCGAAATCCGTTCGGTGAGAGC-3' (Umina et al. 2007)

OFFICIAL

Dvit5_R 5'-TATGGTCAATGGTCAATCCGTC-3' (Umina et al. 2007)

Tail B (VIC) (universal fluorescently labelled primers) 5'-VIC-GCCTTGCCAGCCCGCG-3' (Blacket et al. 2012)

Primers for (NED) Multiplex 4: (Dvit2:Dvit3, 2:1 relative primer ratios)

Dvit2_F_Tail_C 5'-CAGGACCAGGCTACCGTGGCTTAATTTTGTGTCTCAAGTTA-3' (Corrie et al. 2002)

Dvit3_F_Tail_C 5'-CAGGACCAGGCTACCGTGCCAAAACAACCAAGATTTTCTCC-3' (Corrie et al. 2002)

Dvit2_R 5'-TAATGCTTCGTTTTCTAAGTGC-3' (Corrie et al. 2002)

Dvit3_R 5'-GATCCAAACTATGACAAACACCC-3' (Corrie et al. 2002)

Tail C (NED) (universal fluorescently labelled primer) 5'-NED-CAGGACCAGGCTACCGTG-3' (Blacket et al. 2012)

Master Mix:

Each 10µl PCR reaction consisted of:

5µl of 2 x Qiagen Multiplex PCR Master Mix; 1µl of 10 x mix of Reverse primers (containing 2-4µM of each R primer*); 0.5µl of 10 x mix of tailed Forward primers (containing 2-4µM of each F primer*); 1µl of the appropriate fluorescently labelled universal primer (4µM stock) corresponding to tailed forward primer; 0.5 µl of RNAse free water; 2µl of template DNA.

(Note: Initial PCR trials involving the addition of Q-Solution resulted in the loss of some loci from multiplex reactions, Q-Solution was therefore not used).

Primer volume ratios:

To make 100µl of each primer multiplex mix:

**Primer Mix**	**Primer 1 (10µM stock)**	**Primer 2 (10µM stock)**	**Primer 3 (10µM stock)**	**Water**	**(Total)**
FAM-F (1:2:1 ratio)	Dvit4_F_Tail_A 20 µl	Dvit6_F_Tail_A 40 µl	DVSSR4_F_Tail_A 20 µl	20 µl	(100 µl)
FAM-R (1:2:1 ratio)	Dvit4_R 20 µl	Dvit6_R 40 µl	DVSSR4_R 20 µl	20 µl	(100 µl)
PET-F (1:2 ratio)	Dvit1_F_Tail_D 20 µl	DvVSSR3_F_Tail_D 40 µl	-	40 µl	(100 µl)
PET-R (1:2 ratio)	Dvit1_R 20 µl	DvVSSR3_R 40 µl	-	40 µl	(100 µl)
VIC-F (single locus)	Dvit5_F_Tail_B 40 µl	-	-	60 µl	(100 µl)
VIC-R (single locus)	Dvit5_R 40 µl	-	-	60 µl	(100 µl)
NED-F (2:1 ratio)	Dvit2_F_Tail_C 40 µl	Dvit3_F_Tail_C 20 µl	-	40 µl	(100 µl)
NED-R (2:1 ratio)	Dvit2_F_Tail_C 40 µl	Dvit3_F_Tail_C 20 µl	-	40 µl	(100 µl)

Allele Bin Sizes (scored in Geneious, version R11)

Sizes are in base pairs; lower size -PQ788553 upper size "allele name"

Dvit1, 2 peaks expected, 2bp repeat unit, 143 to 155bp range (* Note, no bp change for platform differences).

144.99 -PQ788553 146.40bp "145"

148.27 -PQ788553 149.65bp "149"

150.43 -PQ788553 152.04bp "151"

152.75 -PQ788553 154.25bp "153"

Dvit2, 2 peaks expected, 2bp repeat unit, 270 to 305bp range (* Note, add 3 bp to final size to account for platform differences).

272.28 -PQ788553 274.47bp "273"

275.05 -PQ788553 277.22bp "275"

302.58 -PQ788553 304.64bp "303"

Dvit3, 2 peaks expected, 2bp repeat unit, 183 to 209bp range (* Note, add 2 bp to final size to account for platform differences).

189.01 -PQ788553 191.75bp "191"

206.20 -PQ788553 208.60bp "206"

Dvit4, 2 peaks expected, 3bp repeat unit, 165 to 181bp range (* Note, add 3 bp to final size to account for platform differences).

167.08 -PQ788553 168.47bp "168"

170.17 -PQ788553 171.50bp "171"

175.28 -PQ788553 177.17bp "176"

179.30 -PQ788553 180.50bp "180"

Dvit5, 2 peaks expected, 1bp repeat unit, 140 to 155bp range (* Note, add 3 bp to final size to account for platform differences).

140.98 -PQ788553 141.88bp "141"

142.86 -PQ788553 143.63bp "143"

143.86 -PQ788553 144.62bp "144"

144.75 -PQ788553 145.67bp "145"

151.45 -PQ788553 152.32bp "152"

152.43 -PQ788553 153.22bp "153"

153.37 -PQ788553 154.06bp "154"

Dvit6, 2 peaks expected, 3bp repeat unit, 210 to 225bp range (* Note, add 3 bp to final size to account for platform differences).

211.11 -PQ788553 212.24bp "211"

216.64 -PQ788553 218.41bp "217"

219.41 -PQ788553 221.10bp "220"

222.75 -PQ788553 224.54bp "223"

DVSSR3, 2 peaks expected, 2bp repeat unit, 253 to 303bp range (* Note, no bp change for platform differences).

253.21 -PQ788553 255.52bp "254"

274.60 -PQ788553 276.08bp "275"

297.76 -PQ788553 299.58bp "299"

DVSSR4, 2 peaks expected, 2bp repeat unit, 254 to 272bp range (* Note, no bp change for platform differences).

263.04 -PQ788553 264.80bp "264"

265.68 -PQ788553 267.05bp "266"

267.71 -PQ788553 269.11bp   "268"
269.53 -PQ788553 271.04bp   "270”

<u>**References**</u>

Blacket M.J., Robin C., Good R.T., Lee S.F., & Miller A.D. (2012) Universal primers for fluorescent labelling of PCR fragments – an efficient and cost-effective approach to genotyping by fluorescence. Molecular Ecology Resources **12,** 456–463

Corrie A.M., Crozier R.H., Van Heeswijk R., & Hoffmann A.A. (2002) Clonal reproduction and population genetic structure of grape phylloxera, *Daktulosphaira vitifoliae,* in Australia. *Heredity* **88,** 203-211.

Lin H., Walker M.A., Hu R., & Granett J. (2006) New Simple Sequence Repeat Loci for the Study of Grape Phylloxera (*Daktulosphaira vitifoliae*) Genetics and Host Adaptation. *American Journal of Enology and Viticulture* **57,** 33-40.

Umina P., Corrie A., Herbert K., White V., Powell K.S., & Hoffmann A. (2007) The use of DNA markers for pest management-clonal lineages and population biology of grape phylloxera. *Acta Horticulturae* **733,** 183 - 195.

Vorwerk, S.; Forneck, A. (2006) Reproductive Mode of Grape Phylloxera (*Daktulosphaira vitifoliae*, Homoptera: Phylloxeridae) in Europe: Molecular Evidence for Predominantly Asexual Populations and a Lack of Gene Flow between Them. *Genome* **49**, 678–687.
